# Hypothalamic Hamartomas: Evolving Understanding and Management

**DOI:** 10.1212/WNL.0000000000012773

**Published:** 2021-11-02

**Authors:** Nathan T. Cohen, J. Helen Cross, Alexis Arzimanoglou, Samuel F. Berkovic, John F. Kerrigan, Ilene Penn Miller, Erica Webster, Lisa Soeby, Arthur Cukiert, Dale K. Hesdorffer, Barbara L. Kroner, Clifford B. Saper, Andreas Schulze-Bonhage, William D. Gaillard, on behalf of the Hypothalamic Hamartoma Writing Group

**Affiliations:** From the Center for Neuroscience Research (N.T.C., W.D.G.), Children's National Hospital, The George Washington University School of Medicine, Washington, DC; UCL NIHR BRC Great Ormond Street Institute of Child Health (J.H.C.), Member of ERN-EpiCARE, London; Great Ormond Street Hospital for Children (J.H.C.), NHS Trust, London; Young Epilepsy (J.H.C.), Lingfield, Surrey, UK; Department of Pediatric Clinical Epileptology (A.A.), Sleep Disorders and Functional Neurology, Member of ERN-EpiCARE; HFME (A.A.), Hospices Civils de Lyon, France; Epilepsy Research Unit (A.A.), Barcelona's Children Hospital San Juan de Dios, Member of the ERN EpiCARE, Spain; Epilepsy Research Centre (S.F.B.), University of Melbourne, Australia; Division of Pediatric Neurology (J.F.K.), Barrow Neurological Institute at Phoenix Children's Hospital; Hope for Hypothalamic Hamartomas (I.P.M., E.W., L.S.), Phoenix, AZ; Epilepsy Surgery Program (A.C.), Clinica de Epilepsia de Sao Paulo, Brazil; Department of Epidemiology (D.K.H.), Columbia University Medical Center, New York, NY; RTI International (B.L.K.), Rockville, MD; Department of Neurology (C.B.S.), Beth-Israel Deaconess Medical Center, Harvard Medical School, Boston, MA; and Epilepsy Center (A.S.-B.), Faculty of Medicine, Medical Center–University of Freiburg, University of Freiburg, Germany.

## Abstract

Hypothalamic hamartomas (HH) are rare, basilar developmental lesions with widespread comorbidities often associated with refractory epilepsy and encephalopathy. Imaging advances allow for early, even prenatal, detection. Genetic studies suggest mutations in *GLI3* and other patterning genes are involved in HH pathogenesis. About 50%–80% of children with HH have severe rage and aggression and a majority of patients exhibit externalizing disorders. Behavioral disruption and intellectual disability may predate epilepsy. Neuropsychological, sleep, and endocrine disorders are typical. The purpose of this article is to provide a summary of the current understanding of HH and to highlight opportunities for future research.

## Introduction

Hypothalamic hamartomas (HHs) are deep-seated developmental lesions that have varied manifestations. This review is based on the 4th International Symposium on Hypothalamic Hamartomas, which took place September 12–14, 2019, in Washington, DC, and current concepts, experience, and research. The reader is provided a key clinical summary of HH as well as the most current recommendations for workup and diagnosis. We summarize current thinking regarding pathophysiology, genetic underpinnings, and broad comorbidities of the disease. The surgical management of HH is reviewed. Finally, future research and clinical priorities are considered.

## Search Method

We conducted a thorough search of the literature through PubMed with the search phrase “hypothalamic hamartoma,” identifying 770 articles, 111 of which were published from 2018 to 2021, which were reviewed for content. The subsection editors also reviewed references in their respective sections in regard to their command of material and PubMed. We have included in our references the major publications from the past 3 years and major publications prior to 2018. Where relevant, we listed the most recent article or review (especially for the surgical literature) and we recognize this is often at the expense of the original work or observation. Those important references will be found in the articles' citations.

## Clinical Summary

HHs are lesions of varying size that arise in the ventral hypothalamic region. They are commonly associated with diverse neurologic, endocrinologic, cognitive, behavioral, and psychiatric comorbidities, including epilepsy, epileptic encephalopathy, precocious puberty, and rage. There are 2 classic clinical phenotypes of HH, intrahypothalamic and parahypothalamic, and each is associated with a typical clinical spectrum. Intrahypothalamic HH is associated with epilepsy with gelastic and other seizure types and is often pharmacoresistant. The HH syndrome may include the evolution of developmental regression, psychiatric and behavioral comorbidities, and precocious puberty.^1^ Parahypothalamic HH is usually only associated with endocrinopathy, such as precocious puberty. Epilepsy and cognitive and behavioral disturbances can occur, although rare.^2^

The prevalence of HH is estimated to be 1 in 200,000 children.^3^ The epilepsy syndrome is often associated with the occurrence of gelastic seizures, which are episodes of uncontrollable mirthless laughter, often unrecognized in the early years. These can evolve to include focal seizures with impaired awareness, dacrystic (crying), atypical absence, tonic, atonic, and generalized tonic-clonic seizures, as well as infantile spasms.^4,e1,e2,e3^ Age at onset is typically less than a year for gelastic seizures and between 2 and 7 years of age for additional focal seizure types, both of which are often refractory to medical treatment. Due to the variable clinical presentation, the actual prevalence of pharmacoresistant epilepsy in HH is unknown^5^ but has been reported in various series between 50% and 100%; this high prevalence may represent selection and referral bias.^6,e4^ Rarely, the disorder can be mild and escape diagnosis with symptoms such as “a pressure to laugh” in association with normal cognitive and social development.^7^

The diagnostic evaluation of HH remains challenging. Due to the depth of the lesion, scalp EEG may be falsely normal without evidence of interictal abnormality; ictal surface EEG can be difficult to localize or may be misleading.^8^ In one study of 133 patients with HH, 75% of 584 captured gelastic seizures (56% of patients) had no ictal EEG change.^9^ The hamartoma can be difficult to identify on imaging due to its small size and location, even by experienced neuroradiologists. A high-resolution 3T brain MRI with epilepsy-specific protocol including thin cut 3D T1-weighted (≤1 mm^3^ voxel), T2-weighted, and fluid-attenuated inversion recovery sequences (minimum 2 planes, 3D better) will reliably identify HHs.^10,e5^ The 2014 Florence consensus of the Pediatric Epilepsy Surgery Task Force of the International League Against Epilepsy recommends ictal/interictal EEG to confirm diagnosis (not for localization) as well as 3D volumetric MRI; PET and SPECT are not recommended given significant false results.^11^ The interpretation of this imaging often requires specific expertise, usually more readily available at skilled pediatric epilepsy surgery centers. HH is associated with a varying comorbidity profile that includes neurodevelopmental, behavioral, and psychiatric disorders. Psychiatric comorbidities occur in more than 50% of children.^12,e6^ Rage attacks, as well as less severe aggressive behaviors and attentional problems, are common.^6,13^ Cognitive impairments have been reported in more than 80% of patients, and these appear to be progressive in half of cases. In view of the apparent relationship between the epilepsy onset and neurocognitive difficulty, the syndrome is considered an epileptic encephalopathy, with increased seizure burden contributing to worse cognitive outcomes. Hamartoma removal or disconnection is qualitatively linked to improved cognitive and behavioral outcomes^14^; single-center studies show quantitative improvements in full-scale IQ in patients undergoing pre- and postoperative neuropsychological testing.^15^

There are several hypotheses about how the encephalopathy may develop. It may represent an ictal, interictal, or combined phenomenon.^16^ The encephalopathy may be mediated by the disruption of structural and functional hypothalamic projections including tegmental, mammillo-thalamo-cingulate, and more distributed networks.^4,e7^ There may be local effects within the hypothalamus and depending on which subnuclei are involved, with resultant endocrinologic or functional disturbances contributing to the encephalopathy, as is known to occur in brain tumor diencephalic syndromes. This has also been shown in PET studies in HH.^e8^

## Pathophysiology and Imaging

The cell of origin of HH and the cause of gelastic seizures remains unknown. HHs develop in a region rostral to the mammillary body. A cell group in this region called the lateral tuberal nucleus (LTN) is hypothesized to be the origin of HH. Neurofibrillary tangles are often found in HH^e9^ by histopathology and also in the LTN in neurodegenerative disorders such as Alzheimer disease. A similar cell group (parvalbumin-containing cells) called PV1 lies in a homologous rodent region and has strong connections to the ventrolateral periaqueductal gray.^17^ PV1 cells are associated with generating species-specific vocalizations such as cackling in monkeys. The overgrowth of LTN-derived neurons (HH) could disinhibit PV1 neurons from their normal anterior hypothalamic inputs and may cause the laughter associated with gelastic seizures.^18^ Due to the deep-seated location of the hypothalamus and the recent frequent use of minimally invasive treatment such as laser ablation, there is a paucity of available tissue for study. Newer techniques may allow for tissue recovery from probes used in ablation, as has recently been demonstrated for surgically implanted stereotactic EEG depth electrodes.^e10^

Imaging allows for early detection of HH with high-resolution fetal MRI. The lumen of the third ventricle is not fully established until 26–28 weeks gestational age, so it is difficult to diagnose HH until the second trimester. A common misdiagnosis at this time are interhypothalamic adhesions, which are horizontally oriented bands that connect the medial walls of the third ventricle.^19^ HH are nonenhancing, may be relatively isodense, and may range in size from 2 to 20 mm.^20^

## Genetics

Advances have been made in the genetic underpinnings of HH. The Sonic hedgehog (Shh) pathway regulates neurogenesis and cell patterning in early hypothalamic development; mutations in this pathway can cause increased proliferation of nearby wild-type cells.^21^ Using whole-exome sequencing, chromosomal microarray, and targeted candidate gene sequencing, somatic mutations of Shh pathway-related genes are discovered in one third of tissue and cell samples of patients with nonsyndromic HH.^22^ Similarly, germline mutations in *GLI3*, a transcriptional activator and repressor of downstream Shh pathway targets, cause Pallister-Hall syndrome (PHS), a form of syndromic HH, which can be associated with polydactyly or syndactyly, bifid epiglottis, imperforate anus, and renal abnormalities.^23,e11^ Patients with PHS have less severe epilepsy and fewer neuropsychiatric problems than those with isolated HH.^24^

The location of variants in the *GLI3* gene is associated with different clinical manifestations: frameshift mutations in the middle third of the gene cause PHS, whereas those in the first or last third of the gene cause a distinct syndrome called Greig cephalopolysyndactyly syndrome (poly- or syndactyly, hypertelorism, macrocephaly), which is not associated with HH. The mechanism of this variable presentation was demonstrated in *Drosophila,* which have a homologous gene pathway to human GLI3. The location of a frameshift mutation in *GLI3* can direct whether the downstream function of the resultant protein is activating or repressing, and thus how a single gene mutation can lead to various phenotypes.^25^

An emerging concept is that HH may also develop from biallelic germline and somatic variants within cilia, including Shh pathway genes. The cellular signaling function of Shh proteins depends on their trafficking by cilia during development.^26^ There is preliminary evidence for this in several cilia genes through genetic analysis of a Japanese HH cohort,^27^ and the recent discovery of a biallelic variant in the cilia and hedgehog gene *SMO* in 7 patients with broad developmental anomalies including HH.^28^ Demonstration of this mechanism in further cilia genes of additional nonsyndromic cases would confirm that HH is a ciliopathy. This mechanism may allow for direct therapeutic targeting.

### Focus on Comorbidities

#### Psychiatric

HH is often complicated by psychiatric disorders, which may occur in up to 60%–80% of pediatric cases.^12^ Rage attacks are a poorly defined yet fundamental element of the psychiatric profile associated with the disease.^e12^ In a cohort of 46 children with hypothalamic hamartoma, almost half demonstrated rage attacks.^12^ These events are thought to be caused by biological difficulties with emotional regulation combined with increased aggressive tendencies; their relationship to epilepsy is unclear. Rage attacks and autism spectrum disorder were overrepresented in the Hope for HH International Comorbidity Survey of more than 250 international patients and families (Soeby and Webster, unpublished data; see below). Aggressive behavior more commonly occurs in male patients, those with intellectual disability, those with younger age at seizure onset, and patients with multiple seizure types.^29^ Other commonly encountered psychiatric comorbidities include oppositional defiant disorder, attention-deficit/hyperactivity disorder (ADHD), as well as anxiety and mood disorders.^30^ The rates of ADHD, anxiety disorders, and mood disorders are similar to those seen in many other childhood onset epilepsies; similarly high rates of depression and anxiety exist in adult patients with HH.^31,32^ There is no clear relationship to Delalande subtype.

### Neurodevelopmental

Children with HH exhibit a variety of cognitive profiles from normal to severe disability. Cognitive dysfunction may be progressive and appears dependent on a number of factors. Neurodevelopmental disability is more common in patients with HH with epilepsy. Studies find more severe cognitive impairment in patients with earlier onset of epilepsy, increased seizure burden, increased number of antiseizure medications, and, for some, larger lesion burden.^29,33,e13-e16^ In a retrospective study of 48 patients with HH-associated epilepsy, half of the patients exhibited executive and 62% had verbal memory dysfunction preoperatively. The majority of patients in this study achieved postoperative improvements in intellectual functioning after interstitial radiosurgery or transcallosal/endoscopic resection.^34^ A study of 88 patients with HH undergoing stereotactic radiofrequency thermocoagulation surgery showed postoperative neuropsychological improved performance in all patients (even in those without significant preoperative neurocognitive dysfunction).^35^ However, the relationship between seizures and cognition is unclear as there are cases where successful surgery with regard to seizure control may not normalize other comorbidities/encephalopathy. Early surgery is thought to minimize the risk of encephalopathy, but this remains untested. Many children with HH have developmental and cognitive impairments that predate the recognition of development of HH-associated epilepsy, which may indicate these effects are mediated by HH interference of normal behavioral and memory circuitry. These issues are further compounded by the development of intractable epilepsy, which adds to the burden of disease.^12^

### Sleep

Patients with HH have refractory epilepsy, which can interrupt sleep, leading to an overall poor sleep quality. A cohort of 41 patients with HH from Freiburg, Germany, had less slow-wave sleep than healthy controls. Increased epileptic spikes during sleep are associated with lower IQ and increased nocturnal seizure burden worsens neuropsychological performance (Jacobs, unpublished data). Slow wave sleep has been demonstrated to be important for memory consolidation in children with other focal epilepsies.^36^ As there are no published reports on the relationship of HH and sleep, the mechanisms underlying sleep disturbance and cognitive dysfunction remain to be elucidated.

### Endocrine

The typical primary preoperative endocrinopathy associated with HH is central precocious puberty,^37^ probably because of the premature pulsatile release of hypothalamic gonadotropin-releasing hormone.^6,37^ Occasionally, hypogonadism or acromegaly can be seen,^e17^ and there is a case report of a HH secreting corticotrophin-releasing hormone, causing elevated serum cortisol levels.^e18^ Most of the other endocrine disorders are less common and occur after treatment (e.g., postsurgical or radiation-induced),^38^ but require surveillance and follow-up. The endocrine manifestations include diabetes insipidus,^38^ growth hormone deficiency,^e19^ central hypothyroidism,^38,39^ and the hypothalamic obesity syndrome (HOS).^38,39^ The diagnostic workup and treatment of these endocrinopathies are in accordance with clinical practice guidelines set forth for diabetes insipidus^e20^ and hypopituitarism^e21^ for patients without HH. Approximately 20% of patients with HH develop the difficult-to-treat HOS (hyperphagia, weight gain). HOS management strategies are based on current childhood obesity guidelines including diet and exercise, drug interventions with octreotide,^40^ metformin, potentially newer obesity drugs, and bariatric surgery.^41^ The etiology of HOS is difficult to identify as there can be premorbid obesity in up to half of patients with HH prior to surgery. A semi-quantitative MRI-based scoring system of damage to hypothalamic regions can help predict, based on postoperative imaging, which patients are at risk of developing HOS after surgery.^42^ Postoperative hypothalamic injury is correlated with HOS after damage to posterior hypothalamic subnuclei (such as the dorsomedial nucleus and the dorsal hypothalamic area). These areas are involved in promotion of energy expenditure: contributions from the dorsomedial nucleus cause release of thyrotropin-releasing hormone from paraventricular nucleus neurons; the dorsal hypothalamic area is involved in thermoregulation, activation of brown fat, and locomotion.^42^

### Quality of Life and SUDEP

Hope for HH undertook an international survey of families including 256 respondents (mean age, 18 years; range, 1–55 years) from more than 20 countries to provide information to inform new areas for research and clinical interventions. In this survey, top family concerns are seizures, cognitive sequelae, future uncertainty, psychiatric and behavioral problems, inability to lead an independent life, safety, and sudden unexplained death in epilepsy (SUDEP). Of 256 respondents, 162 had one or more surgical or radiation treatments: laser ablation (n = 61), endoscopic resection (n = 31), transcallosal resection (n = 30), Gamma Knife (n = 27), radiofrequency ablation (n = 27), orbitozygomatic resection (n = 9), and other (n = 27). About half of children achieved seizure freedom. The majority of caregivers reported sleep disturbances, including excessive daytime fatigue and sleep disruption. Endocrine problems were common: >40% report precocious puberty, with many others experiencing abnormal temperature regulation (40%), hypothyroidism (20%), and other signs/symptoms of hypothalamic-pituitary-adrenal axis dysfunction. Emotional dysregulation is frequent, and a majority of patients register rage attacks or behavioral outbursts. More than 40% of patients received a formal diagnosis of an anxiety disorder. A majority of patients have cognitive issues, including >60% of respondents reporting memory problems, learning disabilities, and developmental delays. In 2019, 3 children from the Hope for HH community died of SUDEP, raising the concern that patients with HH are at an increased risk for SUDEP.

### Surgical and Therapeutic Techniques

Several surgical approaches are used to treat HH-mediated epilepsy depending on the location and size of HH, local expertise, and technology available. Surgical procedures can be classified according to their rationale (resection vs disconnection; image-based vs functional) or type of approach (open vs minimally invasive). Table 1 summarizes the main surgical and therapeutic techniques currently being used to treat HH. Except for radiofrequency stereotactic disconnection, all other procedures available for treatment of HH require some type of HH resection. An image-based approach is preferred although some series showed that neurophysiologic data may affect outcome, which is awaiting validation. Endoscopic and stereotactic approaches are considered less invasive than open procedures. Endoscopic approaches might be considered more invasive compared to stereotactic, although no head-to-head comparison is available. Morbidity is related to number of trajectories during stereotactic procedures. One advantage to open and endoscopic procedures is that they can provide tissue specimens. It is arguable that lack of tissue sampling in laser interstitial thermal therapy (LITT), radiofrequency, focused ultrasound, or radiosurgery procedures might negatively affect patient care, outcome, and research in the future, although it may be possible to recover DNA from cells recovered from HH probes. For example, there are para- and intrahypothalamic hamartomas, which have different associated morbidities. A recent review summarizes the most updated neurosurgical data.^43^

**Table 1:**
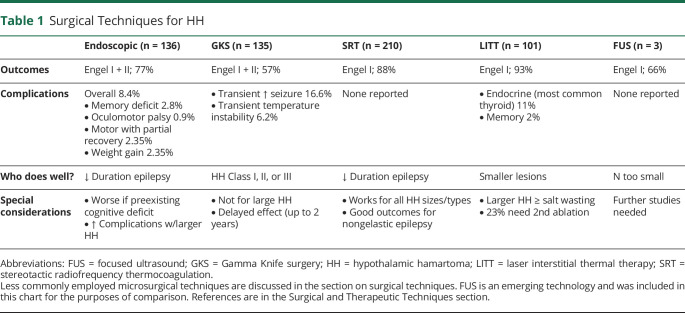
Surgical Techniques for HH

**Figure 1:**
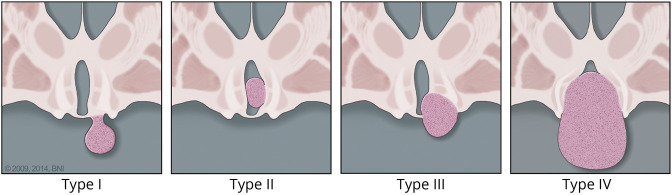
Delalande Classification of Hypothalamic Hamartomas Type I lesions have horizontal attachment inferior to floor of the third ventricle. Type II lesions have vertical attachment to the wall of third ventricle and are above the floor of the third ventricle. Type III lesions have horizontal and vertical attachments above and below the floor of the third ventricle. Type IV lesions are considered “giant” with volume 8 cm^3^ or larger (used with permission from Barrow Neurologic Institute, Phoenix, AZ).

**Figure 2:**
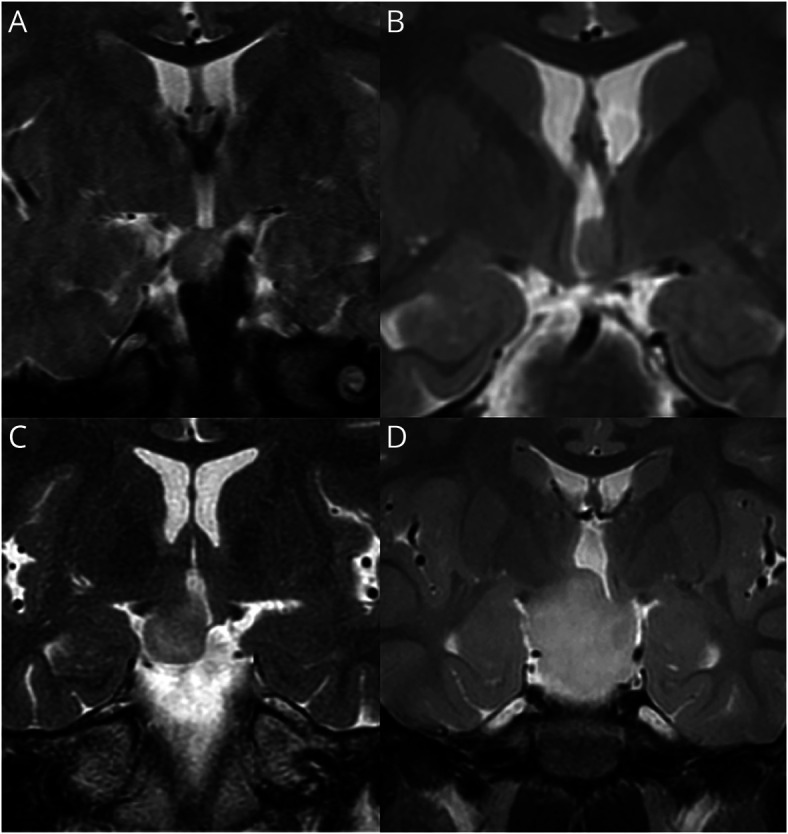
MRI Examples of Delalande Classification of Hypothalamic Hamartoma (HH) All panels show coronal T2-weighted MRI. HH lesion type is designated according to the proposed classification system of Delalande and Fohlen.^44^ (A) Type I HH: 2-year-old boy with mild developmental delay but otherwise asymptomatic. Note attachment of the HH lesion entirely below the floor of the third ventricle. (B) Type II HH: 5-year-old boy with a history of gelastic seizures since 12 months of age. Note attachment to the left side of the hypothalamus, entirely above the floor of the third ventricle. (C) Type III HH: 31-year-old woman with refractory epilepsy, including gelastic seizures since infancy. Note attachment of the HH lesion to the right side of the hypothalamus both above and below the floor of the third ventricle. (D) Type IV HH: 3-year-old girl with severe developmental delay, history of precocious puberty, and multiple daily gelastic seizures. Type IV is designated by Delalande and Fohlen as a “giant” HH, but criteria to distinguish between types III and IV were not offered. We (Barrow Neurologic Institute) currently use a volume measurement over 8 cm^3^ to designate type IV, measuring the maximal length of the lesion in the 3 major axes, then utilizing the formula for determining the volume of an ellipsoid (courtesy of John F. Kerrigan, MD, Barrow Neurologic Institute).

The Delalande classification system groups HH into 4 types based on location and is in turn related to surgical trajectory/approaches and surgical morbidities (Figures 1 and 2).^44^ Endoscopic resection/disconnection can be performed by means of bipolar, radiofrequency, or laser coagulation and achieves a high level of seizure control (up to 78% Engel I + II) but can have an overall complication rate up to 8.3% in a series of 112 patients.^39,e22,e23^ The most common adverse outcomes were transient short term memory deficit (n = 5), oculomotor nerve palsy (transient n = 9, ongoing n = 3), hemiplegia (n = 4), and infection (n = 4). ^e18^ Gamma Knife radiosurgery (GKS) has good long-term safety/efficacy data, but requires a substantial time (up to 2 years) before positive effects are seen, and is not recommended for larger HH.^45,e24^ In a prospective trial of 48 patients with GKS, 68.8% were Engel I + II at last follow-up; no endocrinologic, motor, or amnestic complications were reported.^45^ Stereotactic radiofrequency thermocoagulation (SRT) can be applied to every type/size of HH with significant reduction of gelastic seizure burden.^35,e25,e26^ In a study of 100 patients with HH, SRT led to Engel I outcome in 71% from all seizures types with improved IQ 1 year postprocedure in 69% of the cohort, and reported resolution of behavioral problems in the entire cohort; complications include weight gain, postprocedure precocious puberty, and pituitary dysfunction.^15^ LITT achieves 93% seizure freedom at 12 months^46^; however, it can be associated with salt wasting with treatment of larger HH.^47,e27,e28^ In a series of 18 patients undergoing LITT, complications included weight gain (22%), short term memory loss (22%), and delayed hypothyroidism (11%).^48^ The (surface) laser probe used in endoscopic procedures (distinct from that used during laser interstitial thermal therapy) is applied under direct visualization without intraoperative imaging feedback.^49^ MRI-guided focused ultrasound thermoablation is a newer noninvasive method being studied. There are also open microsurgical techniques including transcallosal resection, orbitozygomatic approach,^50^ transventricular, transcortical, and pterional approaches for the surgical treatment of HH; the orbitozygomatic and pterional approaches may have higher risk than other approaches. There has been a trend away from open microsurgical techniques (such as the original Australian transcallosal approach) to more minimally invasive techniques such as laser interstitial thermal therapy, SRT, and focused ultrasound as success is high and surgical morbidity reduced. In a cohort of 26 patients with HH treated with transcallosal resection, 88% of patients had behavioral improvement; 58% had transient postoperative memory dysfunction and 65% had overall improvement.^38^ A smaller study of 10 patients treated with orbitozygomatic resection, not suitable for lesions below the IIIrd ventricular floor, had 80% improvement in overall quality of life, and 33% of patients experienced improvements in presurgical behavioral and mood problems.^50^ It is postulated that many of the presurgical memory problems relate to HH and proximity to the mammillary bodies; increased anterior–posterior extension around the mammillary bodies is associated with increased risk of cognitive problems.^51^ Surgical techniques, such as endoscopic or microsurgical transcallosal approaches, can incur forniceal contusion, which is related to postoperative memory dysfunction.^52^

A cross-sectional comparative surgical effectiveness feasibility study from 14 international centers of 94 patients (mean 11.8 years, range 1 to 69 years of age) was performed in 2019 on children who underwent epilepsy surgery in 2014–2015. This study did not identify a clear distinction among surgical approaches but demonstrates the need for larger studies and provided evidence of feasibility to that goal (Hesdorffer et al., unpublished data). Prospective collection of short- and long-term adverse effects of various surgical procedures is important as these have likely been underestimated by present studies.

Physiologic characteristics of HH and the nearby hypothalamus that might affect surgical outcome were studied, but the need for an adequate anatomical disconnection of the HH from the surrounding brain was highlighted. Resting-state functional MRI studies identify different connectivity patterns by independent component analysis. These may identify any HH voxel that is functionally connected to other non-HH tissue, which is thought to indicate aberrant and potential pathways for seizure propagation, and correspond to regions of initial ictal propagation by stereo-EEG; their targeted ablation may improve seizure outcome.^53,54^ The unique electrophysiologic characteristics of both the HH and the nearby normal hypothalamus were also studied, and those findings potentially correlated with the area to be resected/disconnected.^55^

Ictal surface EEG may deceptively suggest a cortical focus, which likely represents spread from the HH. Using simultaneous stereo-EEG, multiple seizure types were found to originate in HH with later spread to cortical regions.^56^ Seizure freedom is associated with removal or disconnection of the HH; cortical resection of presumed epileptogenic areas does not achieve seizure freedom.

Human HH slices are being used for in vivo modeling in candidate drug development. One novel therapeutic target is the cannabinoid receptor 2 (CB2), which has been shown to be overexpressed in HH. Using this model, CB2 agonists decreased epileptiform activity and thus may represent a new potential therapy for HH-associated epilepsy (Wu, unpublished data).

## Insights From Other Encephalopathies

There is precedent for molecular treatment of other epileptic encephalopathies, either in tumors from the same region or other genetic syndromes that can show improvement with pathway-specific targeting. Advances in molecular genetics allow for precision therapy of other tumors in the sellar region. For example, there are therapeutic discoveries in low-grade glioma (LGG) with molecular inhibitor therapy targeting the BRAF^V600E^ mutation.^57^ LGG can present as a hypothalamic tumor with a diencephalic syndrome causing encephalopathy that improves or resolves with tumor treatment. Similarly, there are advances in the molecular targeting (everolimus, sirolimus) of the mTOR pathway for the treatment of tuberous sclerosis. While a study in older children of mTOR inhibition failed to demonstrate cognitive improvement,^58^ some investigators hypothesize that mTOR inhibition in young children may help prevent or reverse encephalopathy in tuberous sclerosis complex. Thus, the expansion of the molecular genetics underlying HH may provide similar targeted therapies, and the possibility of improved cognitive outcomes. Such targeted therapies could include *SMO* inhibitors like vismodegib that reduce activation of hedgehog pathway signaling.^59^ Vismodegib is approved for clinical use with demonstrated efficacy for lesion shrinkage or cessation of new lesion development in basal cell naevus syndrome.^e29^ Future prospective studies should incorporate neuropsychological outcomes to formally address this issue.

## Future Directions

### Development of Standardized Management Guidelines

Care for children with HH is not consistent despite center-specific expertise. Standardized pre- and postoperative diagnostic, treatment (including timing and selection of surgical approach), and management guidelines for both children and adults by specialty for neurology, neuropsychology/psychiatry, endocrinology, and imaging will improve care.

Tissue is important for further characterization of somatic mutations and to determine cellular origin of HH. It may be possible to recover HH cells and their DNA from laser ablation probes by adapting a new method developed for stereo-EEG electrodes.^e10^ Furthermore, in vivo models may prove helpful in understanding the pathogenesis of HH. In the rodent models under development, in utero electroporation or tamoxifen is used to transiently induce expression of somatic protein truncating *GLI3* mutations that are known to cause HH in humans (West et al., unpublished data).

Another priority is to characterize a cardinal feature of HH encephalopathy: rage attacks as a distinct feature of HH. A rating/evaluation scale will be important to create and validate for further objective assessment. Another area of focus would be the study of memory dysfunction in HH given the closeness of the hypothalamus to local and remote memory structures (mammillary bodies and more remote memory circuits, e.g., prefrontal cortex).^34^ Other topics of interest include (1) researching the relationship between intellectual or developmental disability, behavior, and seizure control as seizure control may not result in improved behavior; (2) development of pre- and postsurgical psychiatric profiling; (3) better diagnosis/treatment of ADHD/autism spectrum disorder in HH; (4) exploring longitudinal study for psychiatric comorbidity; (5) evaluating support and intervention for managing complex behavioral health issues; and (6) directly comparing the available surgical options.

The question of SUDEP and SUDEP risk needs to be elucidated. Generalized tonic-clonic seizures, a risk factor for SUDEP, are relatively rare in HH. Thus, HH may provide insights regarding SUDEP mechanisms given several anatomical projections from the hypothalamus and functional connection from the HH to the tectum and brainstem structures were recently implicated in SUDEP models.^60^

There is also a need for a coordinated, prospective observational study with standardized assessments and measures to identify the differential effect of therapies on seizure control, encephalopathy, and comorbidities including investigation of early resection/disconnection/ablation on expression of encephalopathy.

## Discussion

HHs are often small, suprasellar masses that can lead to devastating epileptic encephalopathy and comorbidity. The past 2 decades have seen advances in the understanding and management of HH and its comorbidities, with the potential to prevent or reverse the devastating consequences of this disease in some patients. HH presents in 2 ways: the intrahypothalamic variant presents with pharmacoresistant epilepsy (including gelastic seizures), neurocognitive and behavioral sequelae, and endocrine dysfunction. The parahypothalamic variant (seen in syndromic HH such as in PHS) usually causes central precocious puberty, but these patients do not develop severe neurologic comorbidities or epilepsy. The mutations underlying development of syndromic PHS are thought to be in *GLI3*, whereas only a subset of the mutations causing nonsyndromic HH are in *GLI3*, with variants in other hedgehog and cilia genes still being discovered. The exact mechanisms underlying the development of the severe epilepsy syndrome, encephalopathy, and the neurocognitive/behavioral sequelae (including rage) remain elusive. Progress is being made with in vitro and in vivo modeling, molecular genetic analyses, surgical and ablative therapies, as well as broader studies on the neurocognitive and behavioral effects of the disease and its treatment.

## Glossary

ADHD: attention-deficit/hyperactivity disorder
GKS: Gamma Knife radiosurgery
HH: hypothalamic hamartoma
HOS: hypothalamic obesity syndrome
LGG: low-grade glioma
LITT: laser interstitial thermal therapy
LTN: lateral tuberal nucleus
PHS: Pallister-Hall syndrome
SRT: stereotactic radiofrequency thermocoagulation
SUDEP: unexplained death in epilepsy
